# Putting everything in its place: using the INSDC compliant Pathogen Data Object Model to better structure genomic data submitted for public health applications

**DOI:** 10.1099/mgen.0.001145

**Published:** 2023-12-12

**Authors:** Ruth E. Timme, Ilene Karsch-Mizrachi, Zahra Waheed, Masanori Arita, Duncan MacCannell, Finlay Maguire, Robert Petit III, Andrew J. Page, Catarina Inês Mendes, Muhammad Ibtisam Nasar, Paul Oluniyi, Andrea D. Tyler, Amogelang R. Raphenya, Jennifer L. Guthrie, Idowu Olawoye, Gabriele Rinck, Colman O’Cathail, John Lees, Guy Cochrane, Carla Cummins, J. Rodney Brister, William Klimke, Michael Feldgarden, Emma Griffiths

**Affiliations:** ^1^​ Center for Food Safety and Applied Nutrition, U.S. Food and Drug Administration, College Park, MD, USA; ^2^​ National Center for Biotechnology Information, National Library of Medicine, National Institutes of Health, Bethesda, MD, USA; ^3^​ European Molecular Biology Laboratory, European Bioinformatics Institute, Wellcome Genome Campus, Hinxton, UK; ^4^​ DNA Data Bank of Japan, National Institute of Genetics, Mishima, Japan; ^5^​ National Center for Emerging and Zoonotic Infectious Diseases, Centers for Disease Control and Prevention, Atlanta, GA, USA; ^6^​ Department of Community Health & Epidemiology, Faculty of Medicine, Dalhousie University, Halifax, Canada; ^7^​ Faculty of Computer Science, Dalhousie University, Halifax, Canada; ^8^​ Wyoming Public Health Laboratory, Wyoming, USA; ^9^​ Quadram Institute Bioscience, Norwich, Norfolk, UK; ^10^​ Theiagen Genomics LLC, Highlands Ranch, CO, USA; ^11^​ Department of Biology, College of Science, United Arab Emirates University- Al Ain, Abu Dhabi, UAE; ^12^​ Chan Zuckerberg Biohub Network, San Francisco, CA, USA; ^13^​ Science Technology Cores and Services, National Microbiology Laboratory, Public Health Agency of Canada, Winnipeg, Canada; ^14^​ Department of Biochemistry and Biomedical Sciences and the Michael G. DeGroote Institute for Infectious Disease Research, McMaster University, Hamilton, Ontario, Canada; ^15^​ Schulich School of Medicine & Dentistry, University of Western Ontario, London, Ontario, Canada; ^16^​ Faculty of Health Sciences, Simon Fraser University, Burnaby, British Columbia, Canada

**Keywords:** contextual metadata, open data, reproducible science, genome sequencing, public health genomics, pathogen genomics

## Abstract

Fast, efficient public health actions require well-organized and coordinated systems that can supply timely and accurate knowledge. Public databases of pathogen genomic data, such as the International Nucleotide Sequence Database Collaboration (INSDC), have become essential tools for efficient public health decisions. However, these international resources began primarily for academic purposes, rather than for surveillance or interventions. Now, queries need to access not only the whole genomes of multiple pathogens but also make connections using robust contextual metadata to identify issues of public health relevance. Databases that over time developed a patchwork of submission formats and requirements need to be consistently organized and coordinated internationally to allow effective searches.

To help resolve these issues, we propose a common pathogen data structure called the Pathogen Data Object Model (DOM) that will formalize the minimum pieces of sequence data and contextual data necessary for general public health uses, while recognizing that submitters will likely withhold a wide range of non-public contextual data. Further, we propose contributors use the Pathogen DOM for all pathogen submissions (bacterial, viral, fungal, and parasites), which will simplify data submissions and provide a consistent and transparent data structure for downstream data analyses. We also highlight how improved submission tools can support the Pathogen DOM, offering users additional easy-to-use methods to ensure this structure is followed.

## Data Summary

The authors confirm all supporting data, code and protocols have been provided within the article or through supplementary data files.

### Significance as a BioResource to the community

The Pathogen Data Object Model (DOM) will simplify protocols across genomic pathogen surveillance in public health and help standardize the type and structure of metadata submitted to the INSDC. It also sends a strong signal to INSDC developers about where and how to consolidate efforts for improving the data submission/retrieval.

## Introduction

Even as the first large genetic sequence data repositories were beginning to proliferate, issues of curation and data tagging were identified as potential obstacles to progress [1]. These challenges for both contributors and users of these repositories have grown as databases have become larger and more complex. The International Nucleotide Sequence Database Collaboration (INSDC) [2] is a collaborative network of data repositories that have provided public sharing of sequence data for over 40 years. The three organisations that constitute the INSDC are the National Library of Medicine, National Centre for Biotechnology Information (NCBI) in the United States, the European Nucleotide Archive based at the European Molecular Biology Laboratory’s European Bioinformatics Institute (EMBL-EBI) in the United Kingdom, and the DNA Data Bank of Japan (DDBJ). Each member site offers an array of databases, powerful sequence analysis tools, and support services that serve different, yet interlinked, functions. Information is mirrored and mapped regularly across these repositories to provide redundancy and resiliency for the network. However, while the INSDC has become the primary open data repository for pathogen genomic data (rapid public release required now by many major funders), and those data have become essential parts of surveillance, outbreak response, diagnostics, and research, users trying to access the wealth of INSDC pathogen data may be unable to identify important relationships between genomes because the available metadata are often stored inconsistently. As a result, both novice and expert scientists in this field can become confused about how best to structure and submit their data. For example, a submitter can submit an assembled viral genome and minimal contextual data (e.g. the sample metadata, epidemiological, clinical, and laboratory data as well as methods information critical for interpreting the sequence data) directly to NCBI’s GenBank database. Alternatively, EMBL-EBI’s ENA repository offers a more stratified model. Here, someone submitting the same viral genomes would first create a BioProject (called Study or Project at EMBL-EBI), a project-based ‘container’ for collections of datasets, then create individual BioSamples (called Samples at EMBL-EBI), sample contextual data records, and finally attach the raw or assembled genome data. A scientist submitting through DDBJ encounters yet other requirements. Scientists and public health professionals with access to multiple INSDC submission routes may face decision paralysis about which is the ‘best’, ‘most efficient’, or ‘most scientifically productive’ way to submit their data, probably defaulting to whichever seems ‘easiest’ at the time. This default can become limiting as ‘simpler’ flat data organization cannot capture the complex relationships between submitted genomes and the samples from which they were derived. These submission hurdles can be significant, particularly in multijurisdictional global public health investigations or projects. Further, local data protection legislation can lead to a conservative approach to data submission, leaving many potential data types left unshared because of the difficulties in submission, the lack of submitter familiarity with the process, and the barriers of complex technical language and documentation, which are usually only provided in English.

These inconsistencies often result in a patchwork of pathogen sequence and contexual data with no universal method for querying across these different objects. This combination of data variability and complexity adds to the burden on public health laboratories, whose staff are often inexperienced in data management and curation at this scale. While these laboratories may already be overstretched in terms of resources and personnel, they still need to identify and respond to outbreaks in real-time. To demystify submission processes, public health organisations often provide standard operating protocols (SOPs) to their submitters prescribing which databases and submission routes are the most appropriate. However, while SOPs may simplify submissions and data storage for individual organisations, those alone cannot resolve the global problems caused by the many ways and many levels of data provided when people submit sequence and contextual data to the INSDC. That variability impacts how the data can be organized across the network and has downstream consequences for the ways data can be searched, linked, integrated and retrieved by researchers and public health data consumers.

Resolving these global problems requires coordinated action across multiple stakeholder groups. The authors of this paper represent the Public Health Alliance for Genomic Epidemiology (PHA4GE), an international community of public health, research, and industry-based scientists aiming to improve the openness, accessibility, reproducibility and interoperability of public health bioinformatics by establishing global consensus data standards, thereby improving the availability of critical bioinformatic tools and resources, then sharing and advocating best practices.

Here we will formally describe the components of a common pathogen data structure for pathogen data archived within the INSDC, called the Pathogen Data Object Model (DOM), designed to encompass the minimum pieces of data and contextual data/metadata, necessary for general public health utility, while recognizing that submitters will likely withhold a wide range of private health information and sensitive epidemiological data. Also integrated with this data structure are INSDC-developed and third party Graphical User Interfaces (GUIs) and submission tools, which provide indirect entry points into the DOM, abstracting the user from the details of it while still capturing all information necessary to complete the DOM appropriately. Further, we propose this Pathogen DOM to be the default structure used for all pathogen submissions (bacterial, viral, fungal, and parasites). This structure achieves the goal of making data findable, accessible, interoperable, and reusable according to FAIR standards [3] and easily accessible for dashboard monitoring.

There are many reasons why these different submission routes and metadata requirements evolved for the different INSDC repositories. To explain how this diversity developed and demonstrate how our proposed DOM will make research and public health practice more productive, and improve overall data quality and consistency, we provide a brief history of the evolution and network of databases within the INSDC. In each time period, there were great innovations in science, technology, and collaboration, but we also can see how early decisions in data organization and the separation of databases by organism type have contributed to a complicated legacy and some of the important obstacles we see today.

### A brief history of pathogen data sharing within the INSDC

#### Early establishment: 1985 – 2010

The first complete HIV genome was submitted to INSDC in 1985 (https://www.ncbi.nlm.nih.gov/nuccore/X01762), followed by the first complete bacterial pathogen genome, *Haemophilus influenzae,* a decade later [4]. During this time, all sequences, including complete genomes, were made available to the public as ‘flat files’ [5], which was an early INSDC standard structure for communicating features of the sequence data, with contextual data stored as ‘source qualifiers’. By 2009 INSDC was receiving pathogens sequenced on next-generation sequencing (NGS) platforms, reflecting significant technology improvement in the field that led to an explosion of genomic and metagenomic applications.

This same decade saw the formation of the Genomic Standards Consortium [6], providing the first metadata standards for genomic data [7]. A major expansion was also seen at the INSDC, including new databases to hold raw sequence data from NGS platforms (ENA, SRA) and BioProject/BioSample [8] databases to hold project and sample-related contextual data.

#### 2010 – 2019

During this decade, multiple countries started submitting whole genome sequence surveillance data for foodborne pathogens to the INSDC [9–13]. Each submission included both raw reads and a BioSample, using the newly composed pathogen metadata standard implemented for the effort [14, 15]. To eliminate laborious and error-prone manual steps, major public health agencies replaced their manual, browser-based, submissions with automated submissions through NCBI and ENA’s Application Programme Interfaces (APIs). Since independent submitters (e.g. local public health and academic laboratories) generally didn’t have the resources for these API submission pipelines, a suite of detailed submission protocols and best practices were published to explicitly define the pathogen data structure presented here and provide step-by-step instructions for data submission [16, 17]. Older pathogen typing packages, such as BioNumerics (BioMérieux, Inc.), adapted to include NCBI submission for this pathogen data structure, and other third-party submission tools such as METAGENOTE [18] further simplified data submission for academics and public health laboratories.

Real-time data from this effort was being used for timely public health responses [19], which established INSDC, for the first time, as not simply a data repository, but as an essential partner to public health agencies. This utility was supported by the NCBI’s Pathogen Detection browser, providing automated clustering, genotyping screens [20–22] and an interface for querying these results for each pathogen adhering to this submission structure. For the first time, requiring no direct government or public health collaboration, independent submitters who adhered to these standards could submit actionable, interoperable data, and participate in open, global pathogen surveillance. Building on these achievements, multiple third-party data analysis pipelines and dashboards were developed: Innuendo [23], IRIDA [24], Pathogenwatch [25], BV-BRC [26] and others provided dashboards and customized data analyses to public health stakeholder groups.

Through the European Union-funded COMPARE project, EMBL-EBI and partners created pre-publication environments called ‘Data Hubs’ [27, 28] to further lower barriers to sequencing-based pathogen surveillance. Data hubs are linked to ENA submission services, as well as analytical workflows and visualisations, allowing groups of collaborators to rapidly share, analyse and visualize pathogen sequencing data submitted to the ENA prior to public release. Data hubs can be accessed through the ‘Pathogens Portal’ [29], which provides a centralized way to search across multiple pathogens in the INSDC database.

Historically, bacterial and viral resources within NCBI have been managed separately – as each was serving a different group of stakeholders. Much like bacterial genomic data, the first genomic assemblies from viruses were obtained from historic outbreaks and academic research publications. Currently, the NCBI Virus resource [30] makes viral genomic data submitted to GenBank and other NCBI repositories, more accessible, providing users with a single point of entry to easily query both sequence data and metadata (for viruses, metadata can be stored on the assembly flat files and/or in BioSample records). Custom GenBank submission pipelines for Dengue virus, Norovirus, and Influenza A and B virus assemblies eased the burden of GenBank submission for these pathogens.

#### 2020 – 2023: the COVID-19 pandemic

The COVID-19 pandemic marked the first-time genomic sequencing was used for real-time surveillance of a viral outbreak. The first severe acute respiratory syndrome coronavirus 2 (SARS-CoV-2) assembly was submitted directly to GenBank in January of 2020, without a linked BioSample record (GenBank accession MN908947.1). Subsequently, thousands of viral sequence submissions followed this model, routed via NCBI’s GenBank, mostly as consensus sequences, including their metadata (isolate, country, collection date) as source modifiers on the GenBank flat file, rather than creating BioSample records. While all public SARS-COV-2 data submitted to the INSDC is also fed into EMBL-EBI’s dedicated COVID-19 Data Portal [31], part of the COVID-19 Data Platform [32], the differences in metadata models led to confusion within academic and public health labs: what was the most appropriate way to structure pandemic-related submissions?

To resolve this problem, international standards groups, including PHA4GE, made recommendations in 2021 for submitting in the BioProject/BioSample format, and defining what contextual data should be included in a BioSample submission [33]. Public protocols were designed to help submitters adhere to this guidance [34]; however, because there has been no official INSDC agreement or support around this structure for pathogen submissions, a large percentage of INSDC pathogen submissions continued to lack raw data and/or the critical structured metadata necessary for public health interpretation.

#### Moving forward

As this brief history shows, changes in sequencing technologies, the primary stakeholders, genomic data/metadata, the amount of data being collected, and increasing demands for efficient data submission, retrieval, and integration to support the needs of routine and emergency public health operations require a consistent and internationally accepted set of pathogen data standards. Many laboratories across the world have recently acquired genome sequencing capacity for SARS-CoV-2 and have been using custom submission pipelines which were built to encourage rapid submissions of pandemic sequence data. Now these labs are asking questions about how they can expand the scope of their submissions (Box 1): can we start sequencing new pathogen X? What would be the difference between submitting my pathogen sequences to NCBI or ENA? How do I submit my pathogen sequences to one of the INSDC repositories? Do I have to use the Biosample/Bioproject format? What if we can’t provide the full suite of expected metadata?

Box 1: **Community concerns about the BioProject/BioSample data model. A list of commonly raised questions and concerns from the community (laboratorians and data analysts from academia and public health labs) around submitting pathogen sequence data to the INSDC, based on informal information-gathering.**

- How should BioProjects be organized by academics and public health laboratories?
- Should we submit samples that fail quality control?
- Storing contextual data in BioSamples and BioProjects can make the information harder to extract by naïve users (multiple queries required and then the information needs to be linked together) – what do they contribute?
- Submission to the INSDC seems complex. Public health practitioners don’t have the time or interest to learn the more complex aspects and often only have minimal information. How can submissions be simplified?
- Some public health agencies have strict limits on the amount of contextual data they can release publicly. Can they share genomic data while complying with these restrictions?
- What is the protocol for submitting a new species, beyond pathogens under routine surveillance?

Community standards for submitting pathogen data to the INSDC evolved to accommodate the needs of different pathogen communities, resulting in some standardization on how the data are stored within a given pathogen species or class of pathogens. However, significant differences exist between different taxonomic groups such as bacteria and viruses, including where core sample metadata are stored (e.g. on the GenBank flat file for some pathogens and in the BioSample database for others) or which primary genomic data (assemblies or raw reads) are expected for a pathogen species. These inconsistencies can make it difficult for users to build good queries across pathogen types.

In response to this fragmentation, the INSDC members have already built some pathogen-specific resources to help users find what they’re looking for, standardizing some of the pathogen data in the process: EMBL-EBI’s COVID-19 Data Portal [31], NCBI Virus for all viruses [35], and NCBI Pathogen Detection for over 70 bacterial taxa [20, 22]. Using NCBI datasets [36] helps users easily retrieve pathogen genome datasets across multiple resources, such as assemblies, transcripts, proteins, and metadata, no matter how these were originally submitted to the archives. Similarly, EMBL-EBI have recently launched a new and improved version of their ‘Pathogens Portal’ to query multiple data types related to over 200 000 pathogen species [29]. Since EMBL-EBI’s repositories also capture information on human hosts of pathogens, this portal also includes a patient cohort browser connecting different data types across the various archives for each infectious disease cohort study.

These helpful querying interfaces are only as useful as the data inside them, which brings us back to our recommendation of standardizing data across INSDC from the start. The INSDC Pathogen DOM and recommendations we present here provide a set of best practices for improving the consistency and interoperability of open, publicly-available genomics data, and, importantly, these best practices will be applicable across all pathogen types. By controlling the variability in submissions and how associated contextual data is provided, we hope to make better use of current public health surveillance capacity, both for routine and emergency situations. Setting firm criteria for submissions will also simplify training and allow more laboratories to begin to effectively expand their genomic surveillance programmes around the world.

## Methods

**Fig. 1.**
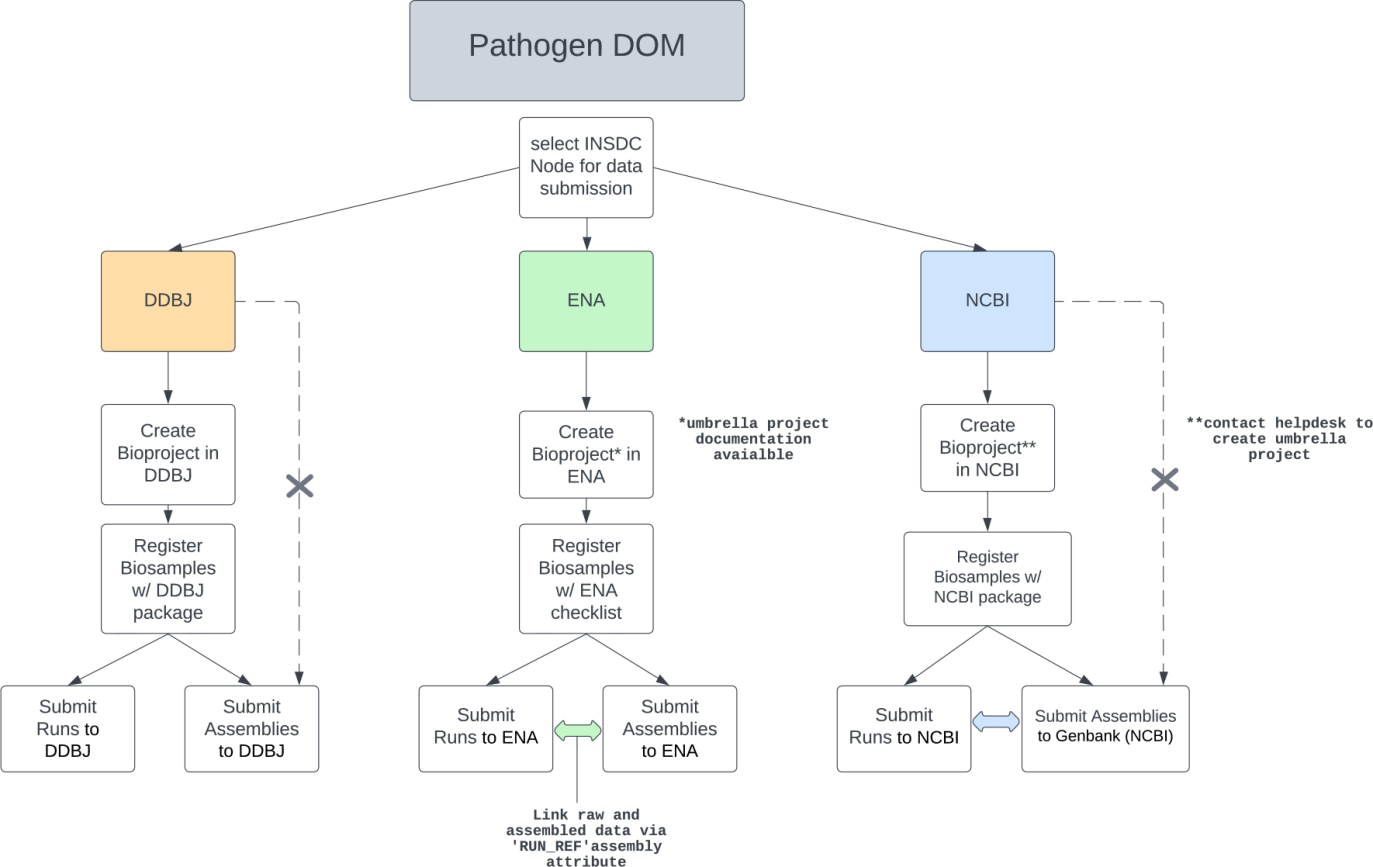
INSDC data structure and main submission paths for each repository. The DOM utilizes existing data structures at each node. Dotted arrows indicate current DDBJ and NCBI allowance for viral pathogen assemblies to be submitted without a Bioproject and/or Biosample. X’s in these dotted lines indicate that these submission pathways are not complient with the Pathogen DOM.

Due to the importance of contextual data accompanying pathogen genome sequence data for public health decision-making, we opted to build upon an existing structured data format within all three INSDC repositories that store these data: BioSample, BioProject, and Raw Read Archive metadata (Fig. 1).

During our informal research and outreach roles working directly with public health and academic laboratories, we found that many submitters and users of INSDC are not aware of why adhering to this structure is so important. This major awareness gap needs to be addressed. Depositing all the raw reads and associated contextual data are essential for effectively querying and analysing complex pathogen data, not just for one laboratory’s immediate needs, but downstream as other investigations need to identify patterns in these data. If this principle is not understood, submitters are likely to avoid what seems a confusing variety of submission requirements for major pathogens across the ENA/NCBI/DDBJ portals, and instead default to the ‘easier’ assembly-only submission routes to GenBank and DDBJ. Doing so, however, means that important contextual data gets embedded within the assembly flat file, rather than in the expected places within BioProjects and BioSamples. Fortunately, once there is agreement within the community and across repositories on a standard structure for pathogen data, third party applications can render submission procedures routine, seamless, and easy for all submitters and users. If implemented properly, users should not need to understand the intricacies of internal database structures in order to submit data or effectively query these public resources.

### Evaluating current state of pathogen data within the INSDC

Evaluating the current state of all pathogen genomes within the INSDC was challenging, since there was no single definition of what a ‘pathogen genome’ is within a repository. For this reason, we accepted the definition of ‘genome’ used by NCBI’s Pathogen Detection, which requires each entry to pass a series of quality control checks (e.g. including expected genome length and other assembly metrics). INSDC data in NCBI Virus were counted as ‘genomes’ if their sequence length met the following thresholds, set by the authors of this study: HIV-1 : 8500–10 000 nt, SARS-CoV-2 : 28 000–30 000 nt, mpox virus: 190 000–200 000 nt. These two *derived* databases import submissions (assemblies, consensus sequence, raw sequence data, BioSamples, BioProjects) from other INSDC databases and therefore have query and quality control thresholds in place for importing data into their respective databases.

## Results

### Describing the INSDC Pathogen DOM

**Fig. 2.**
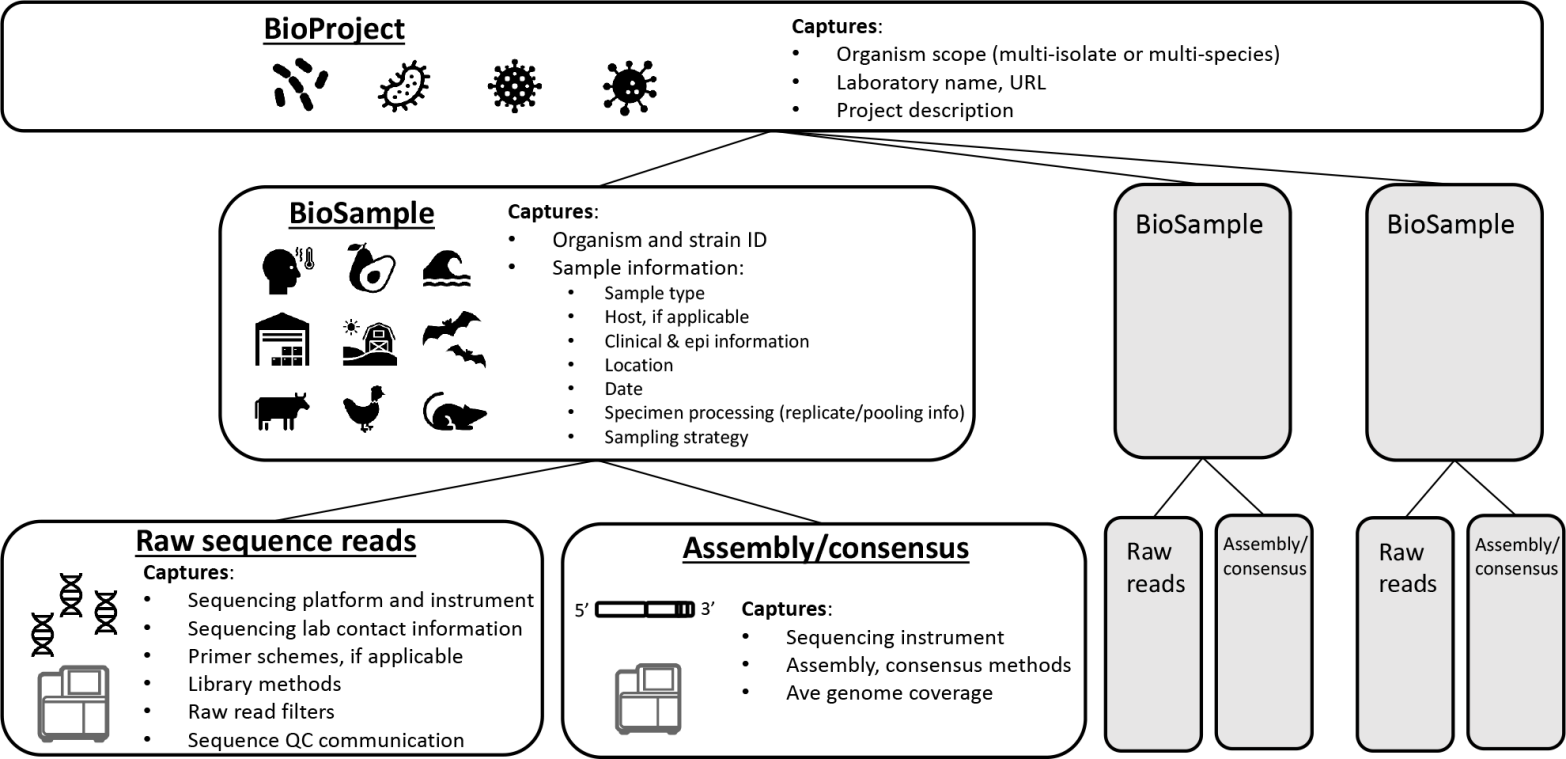
Pathogen Data Object Model (DOM) showing the major components of a complete pathogen package. The primary data pieces of this package comprise the raw sequence files and their associated contextual data stored in BioSample, BioProject, and the raw data archive. Assemblies and/or consensus records are also included in this package and, depending on the organism, can be submitted by the submitter, or generated automatically by the INSDC repository.

To manage complexity and ensure interoperability of pathogen genomic resources, we propose a standard structure for storing *all* pathogen genome data and associated contextual data within the INSDC. This Pathogen DOM has four components: 1) a BioProject for storing project-related information and linking associated data submissions, 2) a BioSample record for providing information about the sample and pathogen, 3) raw sequence data, and 4) an optional assembly/consensus sequence (Fig. 2). A BioProject contains one or more BioSamples, which are linked to raw sequence data and assemblies/consensus sequences. Note that ENA refers to Bioproject and Biosamples as ‘Project or Study’ and ‘Samples’ respectively, but the terms are interchangeable and their structures identical.

BioProject [contextual data]

BioSample [contextual data]

Raw sequence data + contextual data

Assembly/consensus/gene annotations + contextual data (project dependent)

**Fig. 3.**
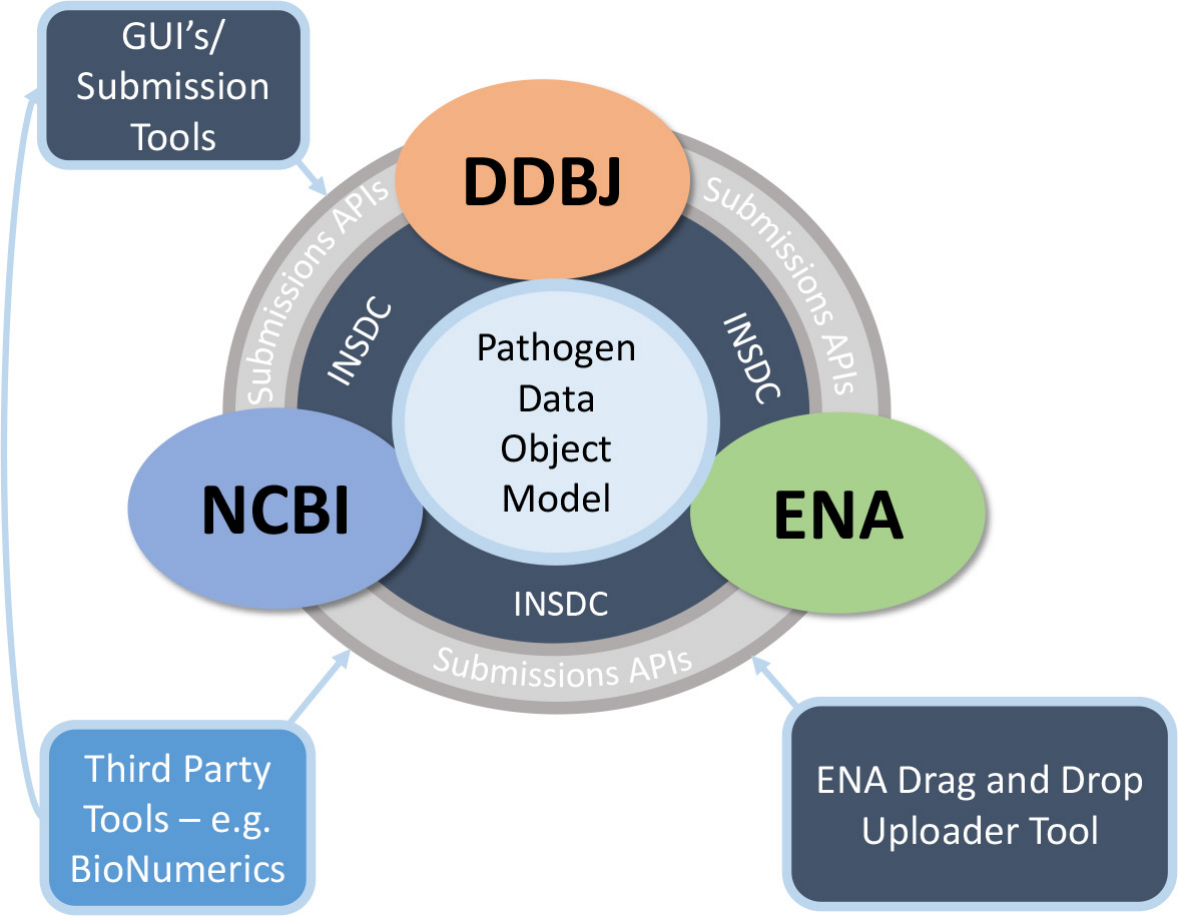
Data flow/entry into the INSDC and subsequently Pathogen Data Object Model. Navy blue boxes represent INSDC developed interfaces and tools, while the light blue box represents external tools, such as BioNumerics or other third party submission tools.

This proposed structure has already had a decade of real-world use by the international enteric pathogen community submitting to the INSDC, demonstrating its utility [9, 17, 37, 38]. Data submissions are easy and can be performed using well-established third party applications such as BioNumerics (BioMérieux Inc.) or by following direct submission protocols specifically written to ensure Pathogen DOM compliance [39]. Recent integrated GUI submission tools, developed during the pandemic, such as ENA’s SARS-CoV-2 Drag and Drop Uploader Tool [40], will further ease submission and conceal the technicalities of the DOM from the user, while still ensuring the underlying data structure is followed in all the respective repositories (Fig. 3). Overall the INSDC-compliant Pathogen DOM provides a standard way of organizing genomic pathogen information within the INSDC, creating a FAIR standard for pathogen sequence data that can support both public health pathogen surveillance and response.

The Pathogen DOM structure has functioned reliably as the quality of sequence data and metadata submissions have evolved and improved over time, while providing a stable core object that can be found reliably within the INSDC and used for routine downstream analyses. New contextual data standards adhering to this model were adapted for SARS-CoV-2 [33], complete with protocols for submitting those data to INSDC [34, 41].

### Solving the contextual information problem

The Pathogen DOM allows important contextual information to be captured at many different levels. *Project-level information* usually includes the name of an academic laboratory, consortium, or public health agency, scope of surveillance or project aims, and funding information. *Sample-level information* might include where and when a sample was obtained; it could also include information regarding sampling strategies, specimen processing, sample types (e.g. anatomical or environmental information, collection devices, and techniques used), host information (e.g. age, gender, health status, symptoms, exposures etc.), diagnostic testing data (e.g. Ct values), pathogen species name, and more. *Raw sequence-level information* will capture how the sequencing process was performed and may include information about library preparation methods and kits, instrumentation, and read processing (e.g. dehosting, primer trimming, low quality read filtering). And, finally, *assembly or consensus-level metadata* can be distilled into one or two pieces of information describing the specific bioinformatic method employed for inferring the sequence. The Pathogen DOM specifies where each of these important pieces of metadata should be stored and how they can be retrieved within the INSDC resources.

### Pathogen DOM components

#### BioProject

In this context, a BioProject is a collection of pathogen sequence records and contextual data submitted by/from a single laboratory, initiative, organization, or consortium. Each BioProject record is accessioned, is accessible via a unique URL, and describes the scope of that specific BioProject. Each BioProject record will provide direct links to its data (BioSamples, raw reads, assembled sequence data) and links can be added to relevant external resources like publications, and funding sources, both of which are critical pieces for academic contributors and their funders.

BioProjects established for pathogen submissions can vary widely in scope to fit the needs of submitters and organizing bodies. Examples of narrow scopes could be species-specific BioProjects established by individual public health labs for each pathogen species they submit. Broad scopes might be comprehensive, multi-species BioProjects that contain data from multiple contributing labs in consortia, or BioProjects from laboratories that share the same funding sources, or other organizing forces.

#### BioSample

A BioSample is a record in a database that stores critical contextual data needed to interpret genomic sequences. BioSamples can link multiple distinct sequence data submissions/experiments to one sample description, e.g. technical replicates, samples sequenced using different specimen processing protocols, or using different sequencing technologies. These linked data allows users to quickly identify different datasets derived from the same sample. Contextual information contained in BioSample can also be queried across projects.

However, rich contextual data also represent one of our biggest challenges: the heterogeneity with which samples and isolates are described. The INSDC offers standardized BioSample attribute packages containing prescribed fields for describing a wide range of sample/isolate characteristics, processes, measurements, phenotypes and data provenance details for the broad range of species for which sequence is generated and submitted. The ENA offers these as ‘checklists’; NCBI and DDBJ call them ‘packages’. In addition, all three repositories offer the Minimum Information about Any Sequence (MIxS) templates [7].

An advantage of using BioSample records to capture and store contextual data about samples and isolates, is that these attribute packages create a framework for structuring sample metadata with mandatory and recommended fields, and can be used in their entirety, or partially, to standardize submitted data. Users can also add additional fields (ideally sourced from other packages or checklists) as needed, to provide flexibility and the ability to customize submissions according to the needs of different pathogens and projects, while providing the standardization and consistency beneficial for public health analyses and analytical platforms. These are important improvements over the ‘Source’ feature in traditional GenBank flat files, which cannot capture the same rich contextual information, and is not as flexible, nor as customizable, as BioSamples.

#### Raw read archive (SRA/ENA/DRA)

While genomic epidemiology and public health surveillance analyses often make use of assemblies and consensus sequences, submissions of raw pathogen sequence data, collections of reads from a sequencing run that may be minimally processed (e.g. removal of human-derived reads), are incredibly important for many reasons. 1) Having the raw data enables many downstream data analyses that cannot be done with assemblies or consensus sequences, e.g. SNP clustering [42], or investigating within-host diversity [43]. 2) Derived data, such as assemblies or phylogenies, depend heavily on what algorithms and tools were used to generate them. Those tools, even widely used ones, may have biases or perform processes that are inappropriate for other uses or in other circumstances. Furthermore, if those algorithms are changed or replaced at a later date, having the raw data is required to re-compute all the derived data downstream. Without the raw starting material, identifying issues with derived data is very difficult. 3) Having the raw data allows genome assembly, or mapping to reference to be automated within the repository, standardizing this step across the whole repository. For these reasons, raw sequence data is an essential component for validation, FAIRness, and quality control, and therefore, highly recommended for inclusion in the submission package.

Contextual data describing sample processing and sequencing are stored with the raw reads. The INSDC provides a generic metadata template for users to populate that structures the information about how the sequence data were generated. In cases where more granular information is needed, user communities have adopted custom attributes to capture crucial components of these methods, such as ‘library_preparation_kit’ and ‘amplicon_PCR_primer_scheme’ developed by PHA4GE for SARS-CoV-2 sequencing methods [33, 41]. Custom attributes can be added to the generic Excel template for NCBI SRA submissions, and ENA also supports custom attributes through their interactive submission route and API.

Contextual data describing the quality of the sequence data can also be included, which can be very helpful for downstream utility. For example, validation studies often require a broad range of sequence data quality, including datasets with low genome coverage, uneven coverage, low-quality sequence reads, shorter than expected read length, varying levels of contamination, etc. Also, during initial phases of method development for emerging pathogens, using these quality control (QC) tags can help public health labs feel more comfortable releasing data before quality thresholds are established for those efforts. A starting suite of QC attributes has already been defined [44] and implemented for SARS-CoV-2 wastewater sequence data [45], but these are broadly applicable to other pathogen surveillance projects.

#### Assembly/consensus sequence database (GenBank/ENA/DDBJ)

Submissions of annotated genome assemblies (the product of a whole genome sequencing workflow) or a consensus sequence (the product of targeted amplicon approaches) provide the community easy access to a lightweight file for many downstream applications. The NCBI Pathogen Detection automates these steps (assembly and GenBank submission) for the submitter, which standardizes the assembly and annotation across the repository and reduces the burden of submission for that derived data type.

For emerging pathogens, sequencing methods are not yet standardized or optimized (different amplicon or enrichment approaches employed, for example), so the content of raw data can vary from submitter to submitter for the same pathogen. In these cases, having the submitter include the derived assembly or consensus sequence in the submission package is crucial for providing the public a lightweight file for downstream analysis (e.g. early SARS-CoV-2 and mpox). Additionally, as part of an ENA submission, assemblies and their derived reads can be linked together via the ‘RUN_REF’ attribute, which specifies the related run accession/s.

### Best practices for implementing the Pathogen DOM

Having described the Pathogen DOM, how it resolves existing problems, and what the components are, INSDC and PHA4GE recommends the following best practices, based on input and consensus from the public health bioinformatics and INSDC communities: data submitted to the INSDC should be organized using the INSDC Pathogen DOM, outlined as follows (Fig. 3):

#### BioProject

Establish BioProjects that reflect natural programmes or surveillance efforts within a laboratory or consortium. Use these objects to define the scope of the effort, specify funding sources, and laboratories involved. To simplify downstream processing, we recommend keeping data obtained using similar methods (i.e. WGS from bacterial pathogens, or amplicon sequencing of viruses).

#### BioSample

Choose the appropriate metadata package (NCBI)[46] or checklist (ENA)[47] that best captures the type of sample being submitted (e.g. NCBI’s Pathogen package, SARS-CoV-2 package, or One Health Enteric package) or one of the appropriate ENA pathogen checklists, (e.g. viral pathogen checklist or prokaryotic pathogen checklist) and determine whether any customization is needed (additional attributes can be newly created or re-purposed from other packages).

#### Raw sequence data

Where possible, the primary sequence data submission should be the raw sequencing data files plus a contextual data describing the sequencing methods and information about any pre-processing performed prior to submission. Project leaders should determine whether the generic metadata template provided by INSDC is sufficient or whether a custom template is required to capture additional information about methods. Importantly, both good quality and poor-quality datasets are useful, provided that QC issues are identified. If poor quality datasets are submitted, known quality control issues should be articulated by including standardized QC contextual data tags. The authors recognize that including raw sequence files in the submission package might be an initial barrier in some low-resourced laboratories, where internet bandwidth might not support movement of these large data files. However, in the long term, technical solutions will likely resolve most of these barriers.

#### Assembly/consensus sequence

Requirements for including or not including assembled genomes are mostly project specific. When present, methods used to infer this derived data type should be included with the record.

While the elements of the Pathogen DOM are the same across the three INSDC repositories (Fig. 1), each repository still offers their distinct methods and tools for data and metadata submission. General guidelines on how to submit data to each INSDC repository are given here:

- DDBJ: https://www.ddbj.nig.ac.jp/ddbj/submission-e.html
- ENA: https://ena-docs.readthedocs.io/en/latest/submit/general-guide.html
- NCBI: https://submit.ncbi.nlm.nih.gov/

### How far are we from meeting these Pathogen DOM standards

The currently available combination of data and metadata for the genomes of major pathogens in INSDC varies widely from pathogen to pathogen. Many viral pathogens, including HIV-1, were almost entirely submitted as single nucleotide records (Table 1), and typically do not have BioProjects, BioSamples, and raw sequence data available. In part this is because nearly half of the HIV sequences archived pre-date the inception of BioProject and BioSample. In contrast, 100 % [*n*=571 697] of the genome submissions for the bacterial pathogen, *Salmonella enterica *, already meet the Pathogen DOM standard, having BioProjects, BioSamples, and raw sequence reads; 85 % [*n*=488 235] of these records are also available as assemblies. Genome submissions of pathogens involved in recent, high-profile outbreaks, such as SARS-CoV-2, mpox virus, and *Staphylococcus aureus *, each have a mix of submission types: 82 % [*n*=6 850 374] of SARS-CoV-2 genomes have Biosamples, 70 % [*n*=4 738 148] have raw sequence data, and 100 % [*n*=8 318 267] have consensus sequences (Table 1). One fifth [*n*=1281] of the mpox genomes have BioSamples, and only 14 % [*n*=861] have raw reads submitted. One hundred percent [*n*=107 363] of *S. aureus* genomes have BioSamples, ~77 % [*n*=83 191] have assemblies, but only 70 % [*n*=75 074] of these records include raw sequence data. One hundred percent of the bacterial pathogens are linked to BioProjects, but this is not the norm for viruses where only 1 % of HIV1 genomes, 84 % of SARS-CoV-2 genomes, and 44 % of mpox genomes are linked to a registered BioProject. Generally speaking, the submitters of bacterial pathogens are closer to the goal of making complete, consistent submissions; efforts to expand these successes will require education, collaboration, and a shared commitment to improve the quality of pathogen genomics worldwide.

**Table 1.** *A summary of current INSDC genome submissions for three viruses and two bacterial pathogens*. Viruses: Human immunodeficiency virus 1 (HIV1), Severe acute respiratory syndrome coronavirus 2 (SARS-CoV-2), and mpox. Bacteria: *Salmonella enterica* and *Staphylococcus aureus *. Submissions to NCBI Virus DB were counted as ‘genomes’ if their sequence length fell between the following sequence lengths: HIV-1 : 8500–10 000 nt, SARS-CoV-2 : 28 000–30 000 nt, mpox: 190 000–200 000 nt. Queries were performed on 15 October 2023

Components of current genome submissions	HIV1	SARS-CoV-2	mpox	* Salmonella enterica *	* Staphylococcus aureus *
**NCBI PD or Virus DB Totals**	25 985	8 318 267	6329	571 697	107 363
BioProject	349 (1 %)	6 968 975 (84 %)	2754 (44 %)	571 697 (100 %)	107 363 (100 %)
BioSample	1214 (7 %)	6 850 374 (82 %)	1281 (20 %)	571 697 (100 %)	107 363 (100 %)
Raw reads	143 (0.5 %)	4 738 148 (70 %)	861 (14 %)	541 152 (95 %)	75 094 (70 %)
Consensus/Assembly	25 985 (100 %)	8 318 267 (100 %)	6329 (100 %)	488 235 (85 %)	83 191 (77 %)

### Advantages for submitters, stakeholders, and the INSDC

#### For submitters

Implementing a standard internal data structure at the INSDC for storing public-health related pathogens is the first step toward standardizing the submission pathway for these data. Currently, public health labs must implement and maintain multiple different submission pathways, each tailored to specific pathogens under surveillance, e.g. one for enteric bacteria, one for SARS-CoV-2, and another one entirely for emerging pathogens like mpox. The same questions get asked by laboratory scientists in public health every time they start sequencing a new pathogen – what is the submission process, what metadata are needed, should we create a new BioProject? If so, should we link it to an existing umbrella effort? Are there flags that need to be set for downstream processes, e.g. Pathogen Detection, Human read scrubbing, etc.

All these questions get at the same primary issue – how should my laboratory submit data in a way that contributes to the broader public health effort? How can I make sure the data I submit are interoperable with others sequencing the same pathogen? These critical questions can all be addressed by first standardizing how pathogen data are stored within the INSDC and synchronizing duplicative data submission (GenBank source qualifiers with BioSample attributes). Once the community agrees on this standard, a single submission process for all pathogens is possible for each respective INSDC repository. Third party applications (e.g. BioNumerics for PulseNet) have a role here as well, for interfacing between the laboratory’s information management systems (LIMS) and the INSDC for brokering submissions and tracking accessions and updates. Submitting the complete Pathogen DOM package for bacteria, viruses, and parasites should be, and can be, ‘push button’ easy for public health labs, academics, and other submitters.

#### For users and stakeholders

**Fig. 4.**
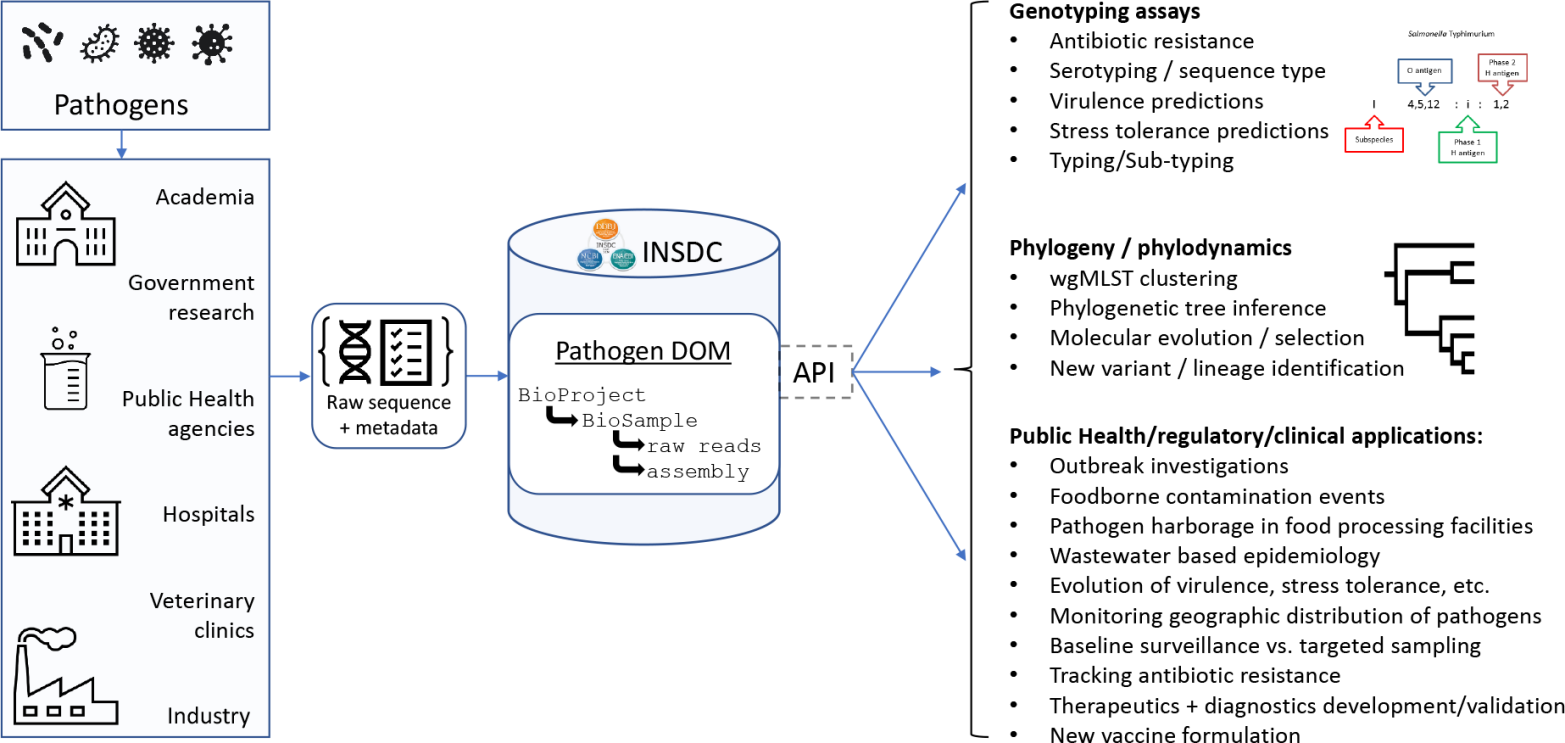
A Pathogen DOM for all pathogens simplifies both submission AND downstream applications and dashboard development. Ideally, a common or standardized application programming interface (API) at each repository would enable easy queries of the Pathogen DOM components.

An implemented INSDC Pathogen DOM for sequence data would provide primary data for feeding all manner of clinical, regulatory, and public health data systems around the world, many of which could hold sensitive patient or contextual information required for on-the-ground decision making (Fig. 4). Using the Pathogen DOM would provide a stable internal data structure for future robust APIs to query data using automated tools (sequence data and contextual data) within these objects. This is crucial for not only downstream applications and dashboard monitoring which rely on having stable objects to query, but also for creating pathogen-agnostic tools – consistent metadata sets could be used to build one dashboard that could be populated with data from any properly-submitted pathogen. The Pathogen DOM also can help academic researchers and students know what they need to find and provide in order to make clear contributions to their fields, and to ensure those contributions are rapidly integrated into relevant public health applications.

Once the Pathogen DOM is implemented more widely, there are potential new stakeholders: groups responsible for regulated testing and enforcement. The INSDC has the potential to become a database of record for official validation purposes (e.g. the Clinical Laboratory Improvements Act [CLIA] in the US) – having standard, versioned data and metadata in an immutable format could become the cornerstone of many sequence-based reference assays. When the question ‘What are your standards for this assay?’ is raised, laboratorians could reply ‘The reference database includes the following set of standardized sequences, which are available in this location.’ Provided these datasets are kept in a standardized structure, regulatory bodies can easily verify and use these data for proficiency testing and enforcement.

#### For INSDC repositories

The Pathogen DOM proposes a single, common data structure for pathogen genome submissions, leveraging existing data structures already in place at all three INSDC repositories. Implementing this standard will greatly improve interoperability of pathogen data between the three INSDC repositories. Additionally, the Pathogen DOM helps integrate resources that already exist within each repository (especially at NCBI and DDBJ), supporting in-house infrastructure and streamlining submission procedures. These improvements would make it easier to create and publish guidance tailored specifically to public health submitters and stakeholders.

Having a standardized structure in place would also improve APIs and cloud query access to pathogen sequence data and associated contextual data, resulting in better third party visualisations, dashboards, analyses [48], risk assessment, etc. And finally, this standard would enable a single linkage point to other heavily used public genomic repositories, like GISAID [49], American Type Culture Collection (ATCC), Genome Portal [50], Joint Genome Institute Genomes OnLine Database (GOLD) [51], and others that hold the same or similar types of data, creating more interoperability between these databases.

## Discussion

Over the last 30+ years, the INSDC has become the world’s largest and most renowned genomics collaboration, hosting a set of shared repositories for sequence and contextual data. Those data, in conjunction with the tools provided to explore them, have revolutionized academic and public health genomics. To maintain the INSDC’s role in empowering public health practitioners to analyse their own data regardless of resource status, it is critical to streamline processes for data submission, data retrieval, and data exploration – in essence, to make it as easy as possible to navigate the system. It has been acknowledged across the community that fragmentation of these processes within the INSDC has led to challenges and uncertainty for users and submitters. In the face of complexity and uncertainty, users often choose the path of least effort and avoid the BioProject/BioSample data model.

Our proposal presents a data model, the INSDC Compliant Pathogen DOM, which leverages existing data structures, resolves uncertainty around how data are best organized and integrated, and sets clear expectations that public health organisations can use to create effective standard operating procedures for their members. This model meets FAIR data standards, and it is already being used by bacterial and some viral pathogen genomics laboratories, demonstrating that Pathogen DOM is workable and generalizable to broad and narrow scopes of work.

What stands between where we are now and where we hope to be? Some barriers – in particular the points requiring decision-making – can be more difficult to address as they are specific to local circumstances. Which contextual data are needed to make sequences more reusable depends on the public health questions being asked, and these, in turn, depend on the public health priorities at the time, as well as the organism or genetic determinants of interest (foodborne pathogen surveillance would likely require different data elements compared to One Health antimicrobial resistance surveillance, which may differ from SARS-CoV-2 wastewater surveillance, etc.). In general, good sample descriptions are usually invaluable (what was sampled, where the sampled entity/host was located, how the sample was collected) as is information about sampling strategies. However, a community-driven data needs assessment which includes justifications and parameters for different data elements may be helpful in providing further guidance in different situations and contexts.

We hope that by articulating the true barriers to progress, which seem to be ‘confusion due to too many options’ and ‘lack of understanding about why all these fields really are important to fill in’, stakeholders can address these needs for clarity, simplicity, and education more directly. For example, PHA4GE has described the benefits of the Pathogen DOM above. While we recognize that implementing this standard might be initially difficult for some, over time, broad adherance to the DOM will simplify protocols across pathogens in public health, and send a strong signal to INSDC developers about where to consolidate efforts for improving the data submission/retrieval. PHA4GE and the INSDC can also work together to make instructional materials more available or to hold and/or promote training sessions. By standardizing a common pathogen data structure within the INSDC, the Pathogen DOM, we hope to bring clarity and stability to how genomic data are submitted, stored, and accessed in the rapidly growing field of pathogen genomics.
